# Determinants of psychological resistance and recovery among women exposed to assaultive trauma

**DOI:** 10.1002/brb3.322

**Published:** 2015-02-28

**Authors:** Heather L Rusch, Erel Shvil, Sarah L Szanton, Yuval Neria, Jessica M Gill

**Affiliations:** 1 National Institute of Nursing Research, National Institutes of HealthBethesda, Maryland, 20892; 2 Henry M Jackson Foundation for the Advancement of Military MedicineMaryland, 20817; 3 New York State Psychiatric InstituteNew York, New York, 10032; 4 Department of Psychiatry, Columbia University Medical CenterNew York, New York, 10032; 5 School of Nursing, Johns Hopkins UniversityBaltimore, Maryland, 21205

**Keywords:** CD-RISC, depression, health, mastery, MDD, optimism, posttraumatic Growth, PTSD, resilience, social support, RRID:rid_000042

## Abstract

**Introduction:**

Women exposed to potentially traumatic events (PTEs) are at high risk for developing psychiatric disorders, including posttraumatic stress disorder (PTSD), general anxiety disorder (GAD), major depressive disorder (MDD), and substance-related disorders. However, this risk is not universal. Most women are resistant (i.e., remain asymptomatic), or recover following a brief symptomatic period. This study examined the psychological factors associated with resistant and recovered outcomes in a sample of high-risk women exposed to assault-related PTEs.

**Method:**

One hundred and fifty-nine women completed the Life Events Checklist and were administered the Structured Clinical Interview for DSM-IV Axis I Disorders. This resulted in three groups: (1) no diagnosis (no past or current psychiatric disorder diagnosis; *n* = 56), (2) past diagnosis (a past psychiatric disorder diagnosis, but none currently; *n* = 31), and (3) current diagnosis (a current diagnosis of one or more psychiatric disorders; *n* = 72). Groups were compared on sociodemographics, PTE exposure, psychopathology, health-related quality of life (HRQOL), and psychological resilience-related factors.

**Results:**

The majority of respondents (79%) did not develop chronic PTSD following assault exposure, and the most common psychiatric outcome was MDD (30%). High endorsement of mastery and social support were associated with the no diagnosis group; and greater reports of mastery and posttraumatic growth were associated with recovery from a past psychiatric disorder. Furthermore, both resilient groups (i.e., no diagnosis and past diagnosis) scored higher on HRQOL measures compared with the current diagnosis group (*P *<* *0.001).

**Conclusion:**

Psychological resilience has ramifications to health and well-being, and identifying these factors has potential to inform preventive strategies and treatment interventions for assault exposed women.

## Introduction

Exposure to a potentially traumatic event (PTE) is common, and approximately 25% of women will report exposure to an assault during their lifetime (Tjaden and Thoennes 2000). A third of these women will subsequently develop one or more psychiatric disorders including, posttraumatic stress disorder (PTSD), general anxiety disorder (GAD), major depressive disorder (MDD), and substance abuse or dependence (Breslau 2009, deRoon-Cassini et al. 2010, Kachadourian et al. 2014). While women exposed to PTEs are at an increased risk for developing psychiatric morbidity, (Kessler et al. 1995, Breslau et al. 1998) most women are resilient and report little or no disruption to their lives (Bonanno and Mancini 2008). Even in assaulted women who initially develop a psychiatric disorder, more than half will recover within 1 year and return to previous levels of functioning (Shalev 2002). Therefore, psychological resilience is common; yet, there are limits in determining which women are resistant or will recover from the initial effects of PTE exposure, and which women will develop chronic symptoms. Moreover, the factors that promote different outcomes of resilience are not fully understood. To address these limitations an improved understanding of the factors associated with trauma resistance and recovery is required to inform preventive strategies and treatment interventions for women exposed to assault.

A more consistent determination of what constitutes a resilient outcome following a PTE is essential in developing an agenda for psychological studies of resilience, due to the varied trajectories following PTE exposure. Therefore, it's critical to differentiate between trauma resistance (i.e., the relative imperviousness to the deleterious effects of stress) and trauma recovery (i.e., the ability to restore homeostasis, which was initially compromised following trauma exposure) (Yehuda et al. 2006). Distinguishing between these resilience-related prototypes, will illustrate the extent to which these constructs are related and distinct. Previously, we developed the “society-to-cells” framework to guide the investigation of factors that influence psychological resilience, and provide a distinction of what constitutes a resilient outcome (Szanton and Gill 2010). In this framework, resilience is determined by one of three outcomes: (1) *resistance*, a state of noncompromised function following challenge, (2) *recovery*, a state of compromised function following challenge, succeeded by a return to previous levels of function, and (3) *rebound*, a state of increased function following challenge (observed in both resistance and recovery outcomes), and also known as posttraumatic growth (Ai and Park 2005). Contrary to these resilient-related trajectories is an outcome of *compromise*, a state of chronic symptoms following challenge that qualifies for a psychiatric disorder diagnosis. This framework integrates other theories of resilience, which refer to resistance and recovery outcomes in terms of hardiness (Kobasa 1979), invulnerability (Rutter 1979), and stress buffering (Haggerty 2012), and a rebound outcome in terms of poststress growth (Aldwin 2009).

Putative resilience factors may be protective in the face of challenge, by contributing to psychopathological resistance; however, the identification of well-established factors may be obscured by heterogeneous samples and diverse trauma characteristics. The most commonly recognized factors associated with psychological resilience are positive coping behaviors (e.g., flexibility, acceptance, and humor), optimism, mastery (i.e., competence and perceived control over one's life), social support, and posttraumatic growth. For example, increased scores on the Connor-Davidson Resilience Scale (CD-RISC), a measure of positive coping behaviors, are associated with reduced risk for PTSD in both men and women (Wrenn et al. 2011). Increased optimism is correlated with psychological resistance across gender and a variety of trauma types (Petros et al. 2013).

While some factors are associated with resistance to trauma effects, others may emerge in effort to recover from and defend against the otherwise long-term impairments associated with stress-related psychiatric morbidity. For example, greater mastery is predictive of recovery from PTSD in African Americans (Alim et al. 2008), and is associated with overall psychological recovery, but not resistance in men and women with high rates of assault (Yehuda et al. 2006). Veterans with clinical and sub-clinical PTSD reported higher levels of posttraumatic growth compared with resistive veterans (Zerach et al. 2013). Taken together, it is essential to examine resistant and recovered outcomes as distinct constructs to determine the psychological factors that promote resistance prechallenge, and the factors that promote recovery in individuals already compromised. In doing so, these findings can facilitate the appropriate implementation of interventions.

**Figure 1:**
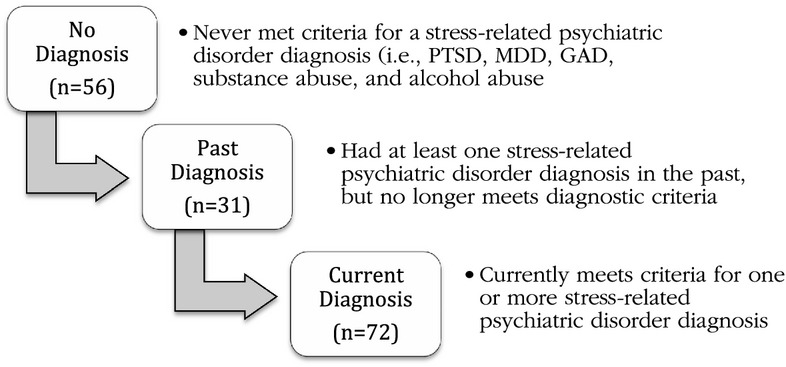
Grouping criteria. PSTD, posttraumatic stress disorder; MDD, major depressive disorder; GAD, general anxiety disorder.

Although there has been considerable effort to determine the psychological mechanisms of trauma resilience, most studies examine resilience within the context of PTSD, and comparatively few studies have examined resilience defined by the absence of a stress-related Diagnostic and Statistical Manual of Mental Disorders (DSM) diagnosis. Surprisingly, there is also a paucity of research examining psychological resilience in women exposed to assault, who are at the highest risk for developing stress-related psychiatric morbidity (Kessler et al. 1995). Moreover, only a few studies have systematically differentiated between resistant and recovered outcomes to distinguish the specific factors that contribute to each resilience outcome. To address this critical gap, we determined psychiatric status in a sample of women with assault-related PTEs, and compared multiple resilience-related measures among the following groups: (1) no diagnosis of a psychiatric disorder, (2) past diagnosis of a psychiatric disorder, and (3) current diagnosis of a psychiatric disorder (see Figure1. for group criteria details). Characterizing these resilience factors may foster the design of novel interventions to prevent psychiatric disorder onset and facilitate recovery from PTE exposure in high-risk populations.

## Method

### Participants

Potential participants were recruited through healthcare provider referral and flyers posted in the emergency department waiting room, the outpatient health clinic, and the community of an academic medical center. Inclusion criteria included women between the ages of 18–60 years old, with exposure to at least one PTE. Exclusion criteria included suicidality and medical instability (e.g., ongoing severe trauma, traumatic brain injury, and need for acute treatment). One hundred and fifty-nine women met eligibility criteria; they provided written informed consent and received monetary compensation in accordance with the Code of Ethics of the World Medical Association and all affiliated institutional review boards (IRBs).

### Methods

Potential participants completed the Life Events Checklist (LEC) (Gray et al. 2004), a self-report inventory of lifetime exposure to various traumatic events. Only those women who reported a DSM-IV: PTSD (Criterion A) assaultive trauma as their index trauma (i.e., most distressful exposure), were included in the study. The Structured Clinical Interview for DSM-IV Disorders (SCID) (First et al. 1996) was used to determine the presence of past and current PTSD, GAD, MDD, and substance and alcohol abuse (collectively referred herein as “psychiatric disorders”). For analysis purposes, this resulted in three groups: (1) no diagnosis (*n* = 56), women who were never diagnosed with a psychiatric disorder, (2) past diagnosis (*n* = 31), women who were diagnosed with a psychiatric disorder in the past, but no longer met diagnostic criteria, and (3) current diagnosis (*n* = 72), women who currently met diagnostic criteria for at least one psychiatric disorder. Eligible participants were also administered multiple resilience-related measures to determine the factors most associated with resistant and recovered outcomes following assault exposure.

### Psychological resilience measurements

The Connor-Davidson Resilience Scale (CD-RISC) (Connor and Davidson 2003) is a 10-item scale that assesses positive coping behaviors over the past month. Higher scores, on a 0–40 scale, indicate greater resilience. The CD-RISC has good internal consistency (0.81), good test–retest reliability (0.85), and excellent validity (0.93) (Campbell-Sills and Stein 2007). The Pearlin Mastery Scale (Mastery-S) (Pearlin and Schooler 1978) is a 7-item scale that measures perception of control over life altering forces. Higher scores, on a 7–35 scale, reflect greater mastery. The Mastery-S has acceptable internal consistency (0.77) (Pearlin et al. 1981). The Life Orientation Test-Revised (LOT-R) (Scheier et al. 1994) is a 10-item test that assesses differences in generalized optimism versus pessimism. Higher scores, on a 0–24 scale, indicate greater optimism. The LOT-R has acceptable internal consistency (0.78), acceptable test–retest reliability (0.79), and adequate validity (Bostock et al. 2009). The Posttraumatic Growth Inventory (PTGI) (Tedeschi and Calhoun 1996) is a 21-item inventory that measures positive changes attributed to a highly stressful event. Higher scores, on a 0–105 scale, reflect more growth. The PTGI has excellent internal consistency (0.90) and acceptable test–retest reliability (0.71) (Tedeschi and Calhoun 1996). Lastly, the MOS Social Support Survey (MOS-SSS) (Sherbourne and Stewart 1991) is a 19-item survey that assesses perception of social network resources on four dimensions. Higher scores, on a 19–95 scale, indicate greater perception of social support. Both total scale and subscales have excellent internal consistency (0.85–0.91) and good test–retest reliability (0.72–0.85) (Sherbourne and Stewart 1991).

### Health-related quality of life measurement

The Short Form Health Survey-36 (SF-36) (Ware and Sherbourne 1992) is a 36-item survey that evaluates patient-reported health-related quality of life (HRQOL) on eight outcomes, including: general health perceptions, energy/fatigue, emotional well-being, role limitations due to emotional difficulties, role limitations due to physical difficulties, physical functioning, social functioning, and bodily pain. Lower scores, on a 0–100 scale, indicate greater disability. Across patient groups, all scales demonstrate excellent internal consistency (0.97), good test–retest reliability (0.85), and excellent validity (0.92) (McHorney et al. 1994).

### Statistical methods

Participants in each of the three groups (i.e., no diagnosis, past diagnosis, and current diagnosis) were classified by age (18–35 vs. 36–60), race (white vs. non-white), marital status (married vs. non married), level of education (some college vs. no college), and household income (less than $40,000 vs. greater than $40,000). The relatively high income split is due to the $65,000 median income in the greater D.C. area (United States. Census Bureau 2012). Omnibus tests of bivariate associations were conducted between the three groups and sociodemographic characteristics, PTE exposure, DSM-IV psychiatric disorder prevalence, HRQOL, and resilience-related factors using two-tailed chi-square tests for categorical variables and one-way ANOVA for nominal and continuous variables (Tables [Bibr b1],[Bibr b2],[Bibr b3],[Bibr b4],[Bibr b5]). Fisher's exact test was used when expected cell counts were less than five. Pairwise comparisons were conducted on variables that were significant at the *P *<* *0.05 level in the omnibus test, using two-tailed chi-square tests for categorical variables and Bonferroni post hoc tests for nominal and continuous variables. All resilience-related factors (Table 5) were entered into multivariate force entry linear regression models, with group status as the dependent variable, to determine the factors most associated with resistant and recovered outcomes compared with a compromised outcome (Table 6). All analyses were conducted using IBM SPSS for Mac, version 21.0 (SPSS, RRID:rid_000042).

**Table 1:** Sociodemographic characteristics.

	I. No diagnosis (*n *=* *56)	II. Past diagnosis (*n *=* *31)	III. Current diagnosis (*n *=* *72)	Omnibus test		
	*n* (%)	*n* (%)	*n* (%)	χ²	*P*	Pairwise^1^
Age^2^						
18–35	28 (50.0)	20 (64.5)	43 (59.7)	2.051	0.359	
36–60	28 (50.0)	11 (35.5)	29 (40.3)			
Race						
White	35 (62.5)	11 (35.5)	30 (41.7)	7.820	0.020	I > II, III
Non-white	21 (37.5)	20 (64.5)	42 (58.3)			
Marital status						
Married	25 (44.6)	19 (61.3)	23 (31.9)	7.876	0.019	II > III
Not married	31 (55.4)	12 (38.7)	49 (68.1)			
Education						
Some college	25 (44.6)	11 (35.5)	25 (34.7)	1.446	0.485	
No college	31 (55.4)	20 (64.5)	47 (65.3)			
Annual income						
<$40,000	30 (53.6)	17 (56.7)	41 (57.7)	0.227	0.893	
>$40,000	26 (46.4)	13 (43.3)	30 (42.3)			

1 Pairwise tests were conducted only when omnibus test was significant at *P *<* *0.05 and are displayed if differences are significant at *P *<* *0.05.

2 Mean age (SD): Resilient 36.7 (9.2), Recovered 32.4 (9.0), Compromised 33.5 (9.1).

**Table 2:** Potentially traumatic event exposure.

	I. No diagnosis (*n *=* *56)	II. Past diagnosis (*n *=* *31)	III. Current diagnosis (*n *=* *72)	Omnibus test		
>	*n* (%)	*n* (%)	*n* (%)	χ²	*P*	Pairwise^1^
Adult physical assault	25 (44.6)	12 (38.7)	50 (69.4)	13.5	0.001	I, II < III
Adult sexual assault	11 (19.6)	8 (25.8)	22 (30.6)	10.1	0.003	I < III
Child physical assault	14 (25.0)	9 (29.0)	39 (54.1)	26.7	0.001	I, II < III
Child sexual assault	20 (35.7)	12 (38.7)	29 (40.3)	3.0	0.704	
Heard about assault	21 (37.5)	18 (58.1)	45 (62.5)	22.7	0.001	I < II, III
Observed assault	10 (17.9)	12 (38.7)	33 (45.9)	18.2	0.001	I < II, III
Death of loved one	20 (35.7)	12 (38.7)	26 (36.1)	0.82	0.792	
Transportation accident	8 (14.3)	6 (19.4)	12 (16.7)	2.8	0.062	
Life-threatening injury	2 (3.6)	2 (6.5)	3 (4.2)	1.9	0.205	

1 Pairwise tests were conducted only when omnibus test was significant at *P *<* *0.05 and are displayed if differences are significant at *P *<* *0.05.

**Table 3:** DSM-IV psychiatric disorder prevalence.

	I. No diagnosis (*n *= 56)	II. Past diagnosis (*n *= 31)	III. Current diagnosis (*n *= 72)	Omnibus test		
	*n* (%)	*n* (%)	*n* (%)	χ²	*P*	Pairwise^1^
Posttraumatic stress disorder						
Current	0 (0.0)	0 (0.0)	34 (47.2)	58.276	0.001	I, II < III
Past	0 (0.0)	20 (64.5)	19 (26.4)	49.838	0.001	I < II, III; II > III
Major depressive disorder						
Current	0 (0.0)	0 (0.0)	47 (65.3)	92.164	0.001	I, II < III
Past	0 (0.0)	18 (58.1)	40 (55.6)	63.291	0.001	I < II, III
Generalized anxiety disorder						
Current	0 (0.0)	0 (0.0)	9 (12.5)	10.358	0.003	I, II < III
Past	0 (0.0)	1 (3.2)	7 (9.7)	6.185	0.039	I < III
Substance abuse						
Current	0 (0.0)	0 (0.0)	12 (16.7)	15.084	0.001	I, II < III
Past	0 (0.0)	5 (16.1)	9 (12.5)	10.649	0.013	I < II, III
Alcohol abuse						
Current	0 (0.0)	0 (0.0)	11 (15.3)	13.472	0.001	I, II < III
Past	0 (0.0)	4 (12.9)	13 (18.1)	13.416	0.004	I < II, III

1 Pairwise tests were conducted only when omnibus test was significant at *p* < 0.05 and are displayed if differences are significant at *p* < 0.01. Fisher's exact test was used when expected cell counts were less than five.

**Table 4:** Health-related quality of life.

	I. No diagnosis (*n *=* *56)	II. Past diagnosis (*n *=* *31)	III. Current diagnosis (*n *=* *72)	Omnibus test		
	*m* (SD)	*m* (SD)	*m* (SD)	*f*	*P*	Pairwise^1^
HRQOL (total)	66.78 (8.10)	58.96 (10.70)	47.05 (13.01)	49.332	0.001	I > II, III; II > III
Bodily pain	68.55 (17.28)	67.94 (11.20)	50.69 (20.28)	17.371	0.001	I, II > III
Emotional well-being	65.23 (13.88)	54.91 (17.33)	41.14 (17.31)	31.972	0.001	I > II, III; II > III
Energy/Fatigue	69.66 (19.18)	49.43 (19.79)	43.97 (23.23)	21.539	0.001	I > II, III
General health perception	64.87 (12.95)	57.76 (11.46)	49.57 (13.89)	19.446	0.001	I, II > III
Physical functioning	64.33 (14.02)	52.67 (16.39)	46.80 (21.76)	13.002	0.001	I > II, III
Role limitations: emotional	64.23 (16.24)	52.48 (15.56)	45.06 (17.83)	18.379	0.001	I > II, III
Role limitations: physical	60.95 (13.88)	58.96 (21.90)	43.67 (22.21)	12.755	0.001	I, II > III
Social functioning	60.94 (18.74)	60.35 (16.76)	54.11 (16.71)	2.572	0.080	–

HRQOL, health-related quality of life.

1 Pairwise tests were conducted only when omnibus test was significant at *P *<* *0.05 and are displayed if differences are significant at *P *<* *0.05.

**Table 5:** Resilience-related factors.

	I. No diagnosis (*n *= 56)	II. Past diagnosis (*n *= 31)	III. Current diagnosis (*n *= 72)	Omnibus test		
	*m* (SD)	*m* (SD)	*m* (SD)	*f*	*P*	Pairwise^1^
Mastery	79.20 (10.41)	80.18 (7.66)	58.12 (17.98)	46.056	0.001	I, II > III
Optimism	63.73 (16.59)	61.64 (15.54)	47.82 (16.61)	17.003	0.001	I, II > III
Posttraumatic growth	61.67 (19.63)	70.88 (18.06)	50.31 (19.12)	13.353	0.001	I, II > III
Social support	66.26 (16.57)	58.03 (17.29)	50.64 (16.28)	13.764	0.001	I > III
Positive coping behaviors (total)	69.37 (14.74)	61.36 (16.05)	49.47 (19.05)	21.748	0.001	I, II > III
1. Adapt to change	4.12 (0.90)	3.55 (1.12)	3.00 (1.33)	14.849	0.001	I > III
2. Deal with whatever comes	3.70 (1.17)	3.19 (1.25)	2.99 (1.22)	5.475	0.005	I > III
3. See humor in problems	3.29 (1.12)	3.19 (1.40)	2.73 (1.09)	3.970	0.021	I > III
4. Cope with stress	3.36 (1.26)	3.26 (1.39)	2.74 (1.10)	4.454	0.013	I > III
5. Bounce back after hardships	4.00 (0.95)	3.68 (1.17)	3.04 (1.29)	11.079	0.001	I, II > III
6. Achieve goals despite obstacles	3.39 (1.26)	3.19 (1.33)	2.79 (1.15)	3.976	0.021	I > III
7. Stay focused under pressure	3.39 (1.19)	3.19 (1.28)	2.85 (1.05)	3.686	0.027	I > III
8. Not discouraged by failure	4.00 (0.93)	3.81 (1.05)	3.00 (1.27)	13.724	0.001	I, II > III
9. See self as a strong person	3.75 (0.98)	3.06 (1.03)	2.70 (1.17)	14.969	0.001	I > II, III
10. Handle unpleasant sensations	3.98 (0.94)	3.48 (0.96)	2.90 (1.40)	13.697	0.001	I > III

1 Pairwise tests were conducted only when omnibus test was significant at *P *<* *0.05 and are displayed if differences are significant at *P *<* *0.05.

**Table 6:** Predictors of outcomes following assault exposure.

	*β*	*t*	*P*
Comparing no diagnosis to current diagnosis			
Mastery	−0.359	−3.958	0.001
Optimism	−0.126	−1.423	0.158
Posttraumatic growth	−0.041	−0.540	0.590
Social support	−0.226	−3.017	0.003
Positive coping behaviors	−0.157	−1.715	0.089
Comparing past diagnosis to current diagnosis			
Mastery	−0.388	−3.976	0.001
Optimism	−0.188	−1.878	0.064
Posttraumatic growth	−0.267	−3.029	0.003
Social support	−0.092	−1.095	0.276
Positive coping behaviors	0.070	0.676	0.500

## Results

### Sociodemographic characteristics

Participants were between the ages of 18–58 years old, with an average age of 34.43 (SD 9.24) years (Table 1). The three groups did not significantly differ on sociodemographic variables (i.e., age, education, and annual income), with the exception of race and marital status, which were controlled for in the analysis. Overall, the no diagnosis group was more likely to be Caucasian (compared to the other two groups), and the past diagnosis group was more often married compared with the current diagnosis group, which was more likely to be single or divorced.

### Potentially traumatic event exposure

All participants reported exposure to one or more assault-related index traumas, details are depicted in Table 2. Aside from assault, the most common PTEs reported included death of a loved one (36.8%), transportation accident (16.4%), and life-threatening injury (4.4%). Within the assault-related PTEs, the no diagnosis group had a significantly lower indirect exposure to assault (i.e., hearing about or witnessing) compared with the past diagnosis and current diagnosis groups (all *P*'s<0.01). There was no difference between the three groups on exposure to childhood sexual assault; however, the no diagnosis group had a significantly lower exposure to adult sexual assault compared with the current diagnosis group (*P *<* *0.01). Both the no diagnosis and past diagnosis groups reported significantly lower exposure to child and adult physical assault compared with the current diagnosis group (all *P*'s < 0.01).

### DSM-IV psychiatric disorder prevalence

The most prevalent current psychiatric disorder diagnoses in the current diagnosis group were MDD (65.3%), PTSD (47.2%), substance abuse (16.7%), and alcohol abuse (15.3%) (Table 3). Within the current diagnosis group, there were also high rates of recovery from MDD (55.6%), PTSD (26.4%), and alcohol abuse (18.1%), yet these participants still met diagnostic criteria for at least one other psychiatric disorder. In the past diagnosis group, the most prevalent past psychiatric disorder diagnoses were PTSD (64.5%), MDD (58.1%), and substance abuse (16.1%).

### Health-related quality of life

The current diagnosis group reported significant deficits on all dimensions of HRQOL, with the exception of social functioning, when compared with the no diagnosis and/or past diagnosis groups (all *P*'s < 0.001) (Table 4). Compared with the no diagnosis group, the past diagnosis group also exhibited significantly lower HRQOL scores in areas of emotional well-being, energy/fatigue, physical functioning, and role limitations due to emotional difficulties (all *P*'s < 0.001).

### Resilience-related factors

The no diagnosis and past diagnosis groups scored significantly higher on measures of mastery (Mastery-S), optimism (LOT-R), positive coping behaviors (CD-RISC), and posttraumatic growth (PTGI) compared with the current diagnosis group (all *P*'s<0.001) (Table 5). The no diagnosis group had significantly higher scores on reported social support (MOS-SSS) compared with the current diagnosis group (*P *<* *0.001); however, this relationship was not observed between the past diagnosis and current diagnosis groups (*P *>* *0.05).

### Resilience-related factors associated with no diagnosis and past diagnosis outcomes

Multivariate force entry linear regression models were used to determine the resilience-related factors most strongly associated with no diagnosis and past diagnosis outcomes (Table 6). The significant predictors of a no diagnosis (vs. current diagnosis) outcome were mastery (*β *= −0.359, *t *= −3.958, *P *<* *0.001) and social support (*β *= −0.226, *t *= −3.017, *P *=* *0.003), with an overall (*R*^2^ = 0.448, *f*_(5,113) _= 18.374, *P *<* *0.001). The significant predictors of a past diagnosis (vs. current diagnosis) outcome were mastery (*β *= −0.388, *t *= −3.976, *P *<* *0.001) and posttraumatic growth (*β *= −0.267, *t *= −3.029, *P *=* *0.003), with an overall (*R*^2^ = 0.412, *f*_(5,92)_=12.893, *P *<* *0.001).

## Discussion

The primary goal of the current study was to examine the association of psychological resilience factors with DSM-IV psychiatric disorder resistance, as well as recovery in a sample of women exposed to PTEs. All participants were exposed to at least one assault-related PTE; however, the no diagnosis group had significantly less reports of indirect assault. Exposure to indirect trauma can weaken psychological defenses (Shultz et al. 2012, Cieslak et al. 2013), which may have increased the risk for psychiatric morbidity in the past diagnosis and current diagnosis groups. Furthermore, the current diagnosis group reported the highest exposure to adult physical and sexual assault. Therefore, resilience factors may not only buffer the damaging effects of traumatic stress, but may also modulate the probability of subsequent exposure to PTEs, by equipping individuals with the social support and mastery necessary to leave dangerous situations. In a study of women sexual assault survivors, those who were revictimized within 1 year reported use of more maladaptive coping strategies than those who were not revictimized (Najdowski and Ullman 2011). Future studies may examine whether resilience factors contribute to an individual's ability to seek out safe environments and discriminate between safe and threatening stimuli.

While the data clearly revealed that the overriding majority of respondents did not develop chronic PTSD; the most common psychiatric outcome in this sample was MDD. This outcome may be explained by the attribution of responsibility and blame, as well as a sense of betrayal by intimate partners or people known to the assault survivor (Abrahams et al. 2013, Ullman et al. 2014). Self-blaming attributions for assault, have been associated with increased levels of PTSD and MDD symptoms (Hassija and Gray 2012). These findings bring greater awareness to the need for complex interventions that also recognize and address trauma-related self-blame depression.

Repeated exposure to psychosocial stressors has been linked to damaging biological consequences (McEwen 2006) ensuing prolonged activation of allostatic systems (McEwen and Wingfield 2003), which contribute to psychiatric symptom burden (Gill et al. 2005). Stress-related psychiatric disorders can then increase the risk for medical comorbidities that may further affect quality of life (Gill and Page 2006). As such, lower HRQOL indicators in this sample were related to a current psychiatric disorder diagnosis, and these health declines remained (although less severe) in the past diagnosis group. These findings support past studies, which found a negative association between HRQOL and PTSD and MDD symptoms (Pacella et al. 2013). This highlights the need for continued health surveillance following the resolution of stress-related psychopathology. Global symptom severity scores and clinicians’ subjective ratings of general improvement do not identify specific domains of functioning or capture patient-reported coping (Westphal et al. 2011), which would otherwise elucidate alternate target areas for intervention. For example, individuals with obesity (Lopresti and Drummond 2013) and sleep disturbance (Mysliwiec et al. 2013) are at higher risk for a range of psychiatric disorders, which have similar disrupted biological pathways (Pace and Heim 2011, Haroon et al. 2012). Therefore, trauma focused interventions that incorporate a component of HRQOL enrichment, may yield enhanced improvements in PTSD and MDD symptom severity. A nurse-led program, which integrates mental health care with care for other chronic conditions, was effective in reducing anxiety and depressive symptoms, as well as improving HRQOL in the elderly (Markle-Reid et al. 2014).

Although optimism and mastery were significantly and negatively correlated with a current psychiatric disorder diagnosis, only mastery was associated with the no diagnosis and past diagnosis groups in the current sample. These results suggest that the apparent predictive power of optimism found in this study and others (Bostock et al. 2009) may have been derived from its substantial overlap with mastery (Marshall and Lang 1990). Mastery refers to the degree to which an individual perceives control and influence over life circumstances. By contrast, optimism is the expectation of favorable outcomes that are not directly attributable to personal factors (Marshall and Lang 1990). Previous studies link mastery to reduced PTSD onset in up-rooted Israelis (Ben-Zur 2008), attenuated PTSD symptoms in veterans (Potter et al. 2013), and mitigated depression in women with intimate partner violence exposure (Rodriguez et al. 2008). Higher mastery is also related to greater quality of life, as well as reduced diabetes-related distress (Yi et al. 2008) and cardiovascular disease mortality (Surtees et al. 2010). Together these studies illustrate the role of mastery in resisting and recovering from the effects of psychological trauma and health-related stressors. Cognitive behavioral therapy (CBT), a widely employed treatment for traumatized populations, promotes greater mastery by helping patients regulate distressing thoughts and emotion. Mindfulness interventions, which strengthen equanimity to the present moment, also foster mastery and have been shown to improve PTSD and MDD symptoms, alcohol problems, and physical health issues following trauma exposure (Smith et al. 2011, Kearney et al. 2012, Kearney et al. 2013). However, low mastery is linked to both noninitiation of therapy and lack of treatment completion (Kegel and Fluckiger 2014). Therefore, any resilience building initiative requires an initial identification of patients with impoverished mastery, so treatment access and adherence may be facilitated.

Perceived social support, rather than the actual support received, plays an important role in predicting psychological well-being and HRQOL (Cook et al. 2009, Weinberg 2013). In contrast, impaired social support is one of the most powerful risk factors for PTSD vulnerability (Brewin et al. 2000, Ozer et al. 2003), as well as depression (Cruwys et al. 2013, Lindfors et al. 2014). High scores of social support were strongly associated with the no diagnosis group in the current sample. However, perception of social support failed to play a significant role in the past diagnosis group once a psychiatric disorder developed. Unlike the stress-buffering effects that social support networks provide to military personnel (Pietrzak et al. 2009, Goldmann et al. 2012, Tsai et al. 2012), and groups with less stigmatized traumas (Gabert-Quillen et al. 2012, Prihodova et al. 2014), disclosure can have both positive and negative impacts on the recovery process for women exposed to assault (Filipas and Ullman 2001, Ullman et al. 2010). Unsupportive, unreceptive, and critical responses from social resources increased the risk of PTSD in physical and sexual assault survivors (Ullman and Filipas 2001, Andrews et al. 2003), most likely by discouraging open communication, which increases cognitive avoidance and suppression of trauma-related memories (Evans et al. 2013, Ullman and Peter-Hagene 2014). Stigmatization, most relevant to sexual assaults, encourages social withdrawal and confirms maladaptive perceptions of self-blame, which are linked to depression (Filipas and Ullman 2001, Hassija and Gray 2012). Given the pernicious impact these maladaptive attributions have on recovery, cognitive restructuring techniques are valuable in challenging these misconceptions (Hassija and Gray 2012). They've also shown benefits in processing avoidance, as well as enhancing personal resilience, and social functioning in women following an assault (Resick et al. 2002). Stigma reducing community outreach efforts and positive social support resources are critical, so women recovering from assault can too benefit from supportive social relations.

While social support had a significant role in buffering the initial effects of stress in this sample, posttraumatic growth was linked to recovery from a psychiatric disorder in the aftermath of trauma. These findings indicate that posttraumatic growth is not a direct consequence of trauma exposure, but arises from the cognitive struggle for a renewed reality during the assumption-shattering aftershock (Calhoun and Tedeschi 2004). This process involves working through alternating cycles of intrusion and avoidance (Joseph et al. 2012), which may suggest that posttraumatic growth is indicative of psychopathology, but instead these constructs reflect adaptive cognitive processing (Helgeson et al. 2006). In fact, greater posttraumatic stress was associated with greater posttraumatic growth following the September 11, 2001, attacks, but only up to a point, above which posttraumatic growth declined (Butler et al. 2005). This curvilinear relationship was reflected in this sample too, with the highest endorsement of posttraumatic growth in the past diagnosis group and the lowest reports in the current diagnosis group. Posttraumatic growth is not simply a return to baseline function following a symptomatic period; instead it is a multifaceted experience of improved function manifested through a perception of new possibilities, increased sense of connectedness, enhanced personal strength, greater appreciation for life, and deepening in spiritual belief (Zerach et al. 2013). Since posttraumatic growth has been linked to attenuated posttraumatic and depression symptoms (Morrill et al. 2008), as well as increased HRQOL (Morrill et al. 2008), using an affective–cognitive processing model collaboratively with assault survivors can help foster posttraumatic growth and facilitate recovery (Joseph et al. 2012). Engaging the patient in consistent exposure-related activities to encourage trauma reappraisal, normalizing distressing emotional states, and promoting relaxation and gratitude exercises are also helpful techniques for overcoming processing difficulties and promoting growth (Joseph et al. 2012).

Of the demographic variables, Caucasians were most prevalent in the no diagnosis group. Ethnic minority status is often reported as a risk factor for the development of PTSD (Breslau et al. 2004); however, findings are less disparate when confounding variables, such as low socioeconomic status (Bonanno et al. 2007) and utilization of health care services (Price et al. 2014) are taken into account. Post hoc analyses using least squares differences correction for multiple comparisons revealed no significant distinctions by ethnic class. This supports other studies where ethnic differences in PTSD were rendered nonsignificant after socioeconomic factors were statistically controlled (Adams and Boscarino 2005, Bonanno et al. 2007). The current study did not offer insight into the reason why married status was associated with the past diagnosis group. This topic warrants further investigation, as past studies report mixed findings (Kessler et al. 1995, Thomas et al. 2014), which may be attributed to an evolving archetype of marital union, where couples adopt long-term partnerships in lieu of legal marriage, as well as the variability in quality of marital relations, especially in samples where intimate partner violence is prevalent.

The results of the present study should be interpreted in light of the study limitations. The sample size was relatively small, and included only women who sustained an assault. Due to the cross-sectional study design, causal relationships between endorsement of a resilience factor and a psychiatric disorder diagnosis cannot be determined. Larger prospective studies are required to determine which resilience factors are linked to trauma resilience, including assaults. Lastly, the standard operational definition of resilience (i.e., a negative PTSD diagnosis) was broadened to include the absence of a stress-related DSM-IV psychiatric disorder diagnosis. This decision was made based on prior research indicating that assaulted women present with MDD and/or substance-related disorders in lieu of PTSD. Increasing the sensitivity of our grouping criteria may have resulted in the misclassification of some MDD and alcohol/substance abuse cases that manifested spontaneously independent of the assault.”

## Conclusion

In this study, we propose that the term “resilience” refers to the absence of a stress-related DSM-IV psychiatric disorder diagnosis following assault, and that resistant and recovered outcomes are two distinct constructs warranting separate examination. The most common psychiatric outcome in this sample was MDD, which may be attributed to self-blame common to survivors of assault. We also found that mastery and social support were associated with the no diagnosis group, while mastery and posttraumatic growth were related to recovery from a past psychiatric disorder. These findings have significance in promoting health and well-being in women, as well as identifying individuals who are most in need of resilience promoting interventions. Longitudinal studies are needed to clarify the factors consistently associated with resilience in women exposed to PTEs and to determine the extent that these factors may be modified through clinical intervention.
